# Wrapping cytochrome c around single-wall carbon nanotube: engineered nanohybrid building blocks for infrared detection at high quantum efficiency

**DOI:** 10.1038/srep11328

**Published:** 2015-06-11

**Authors:** Youpin Gong, Qingfeng Liu, Jamie Samantha Wilt, Maogang Gong, Shenqiang Ren, Judy Wu

**Affiliations:** 1 Department of Physics and Astronomy, University of Kansas, Lawrence, Kansas, 66045, USA; 2 Department of Chemistry, University of Kansas, Lawrence, Kansas 66045, USA

## Abstract

Biomolecule cytochrome c (Cty c), a small molecule of a chain of amino acids with extraordinary electron transport, was helically wrapped around a semiconductive single-wall carbon nanotube (s-SWCNT) to form a molecular building block for uncooled infrared detection with two uniquely designed functionalities: exciton dissociation to free charge carriers at the heterojunction formed on the s-SWCNT/Cty c interface and charge transport along the electron conducting chain of Cty c (acceptor) and hole conducting channel through s-SWCNT (donor). Such a design aims at addressing the long-standing challenges in exciton dissociation and charge transport in an SWCNT network, which have bottlenecked development of photonic SWCNT-based infrared detectors. Using these building blocks, uncooled s-SWCNT/Cyt c thin film infrared detectors were synthesized and shown to have extraordinary photoresponsivity up to 0.77 A W^−1^ due to a high external quantum efficiency (EQE) in exceeding 90%, which represents a more than two orders of magnitude enhancement than the best previously reported on CNT-based infrared detectors with EQE of only 1.72%. From a broad perspective, this work on novel s-SWCNT/Cyt c nanohybrid infrared detectors has developed a successful platform of engineered carbon nanotube/biomolecule building blocks with superior properties for optoelectronic applications.

Single-walled carbon nanotubes (SWCNTs) have superior photoresponse in infrared (IR) spectrum and hence outstanding potential for nanoscale optoelectronic applications with extraordinary performance in addition to the benefits of low cost, large abundance, and light weight^1^,^2^,^3^,^4^,^5^,^6^,^7^,^8^,^9^,^10^,^11^. However, the photoexcited electron-hole pairs (or excitons) in SWCNTs have an unusually high binding energy on the order of few hundreds meV due to the much enhanced Coulomb interaction and much reduced screening effect characteristic to one-dimensional (1D) systems like SWCNTs, which seriously hinders the excitons dissociation into photocurrents^12^,^13^,^14^,^15^. Implementing an effective exciton dissociation mechanism therefore represents a critical challenge toward high-performance photonic CNTs-based IR detectors. In our recent work, a type-II heterojunction of about 1.3 eV band-edge offset formed at the interface between purified semiconductor SWCNTs (s-SWCNTs) and conjugated semiconductor Poly(3-hexylthiophene) polymer (P3HT) has been illustrated efficient in direct exciton dissociation to free carriers^16^. In this s-SWCNT/P3HT nanohybrid photoconductor, the high photoresponsivity of *R*_*i*_ ~ 2.2 mAW^**−**1^ to near infrared light (NIR), and especially the high detectivity D* of 2.3 × 10^8 ^cm Hz^1/2^W^**−1**^ were shown with improvement by more than an order of magnitude than that of previously reported CNT-based bolometers^17^,^18^,^19^,^20^,^21^ and Schottky devices^11^. While this result is exciting, the external quantum efficiency (EQE) of these devices is below 2% due to difficulties in raising the SWCNTs concentration beyond 3 wt % in P3HT while maintaining well dispersed SWCNTs in the polymer to increase proportionally the NIR absorption. At higher SWCNT concentrations, bundled SWCNTs in the s-SWCNT/P3HT nanohybrid prevent effective formation of heterojunctions and lead to much reduced quantum efficiency and increased dark current^22^. Increasing the s-SWCNT/P3HT nanohybrid thickness may be an alternative resolution, but the benefit is outweighed by the charge recombination at a longer carrier transport path in reaching the electrodes. It is therefore necessary and important to explore more efficient approaches to form heterojunctions to s-SWCNT for higher-performance SWCNT-based photodetectors.

Biological molecules, such as DNA and protein, exhibit a great advantage in forming heterojunctions with SWCNTs due to their long molecular chains and large surface areas, which can enable helical wrapping around an SWCNT via either covalent or noncovalent interactions to form π-stacking onto the side-walls of SWCNT^23^,^24^. Cytochrome complex (Cyt c) is an excellent biomolecule candidate for this purpose. Cyt c is a metalloprotein containing heme groups and is highly soluble in water^25^,^26^. This provides the critical feasibility for Cyt c molecules wrapping around individual s-SWCNTs to form heterojunctions at the interface of an s-SWCNT/Cyt c nanohybrid building block. When stacking these building blocks of 1D morphology into thin films, a high s-SWCNT concentration can be achieved. In particular, as an essential component of the electron transport chain, Cyt c could effectively carry electrical current in the s-SWCNT/Cyt c nanohybrid films with minimal charge recombination^25^,^26^. In this letter, we report NIR responsivity up to 0.77 AW^−1^ and EQE in exceeding 90% in s-SWCNT/Cyt c nanohybrid thin film NIR detectors, in which the high responsivity represents a more than two orders of magnitude improvement over that achieved in their s-SWCNT/P3HT nanohybrid counterparts with EQE of only 1.72%^16^, and hence the best so far achieved in CNT-based NIR detectors. This result suggests the s-SWCNT/biomolecule nanohybrids may provide a promising pathway towards photonic IR detectors with performance surpassing the fundamental limitations of exciton dissociation in 1D nanostructures.

## Results

**Figure 1:**
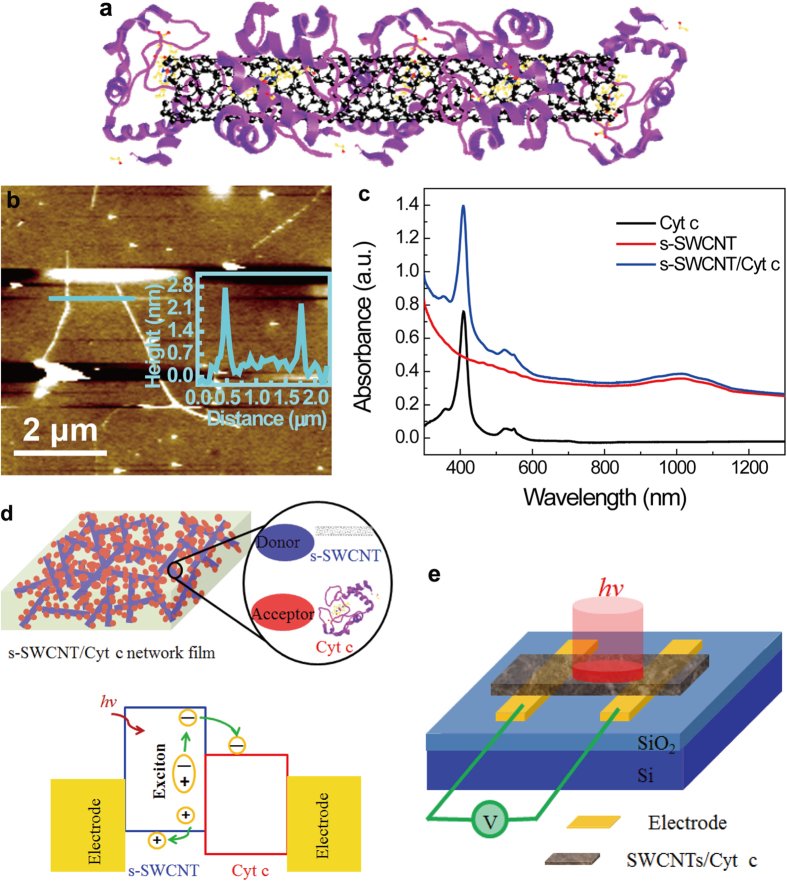
Nanohybrid s-SWCNT/Cyt c photodetector and its operation mechanism. **a**, Schematic of a s-SWCNT/Cyt c building block with Cyt c. **b**, AFM image of individual s-SWCNT/Cyt c building blocks dispersed on a SiO_2_/Si substrate. The inset shows the cross section profiles of two s-SWCNT/Cyt c building blocks under the marked line (cyan) in Fig. 1b. **c**, Optical absorbance spectra of the pure Cyt c, s-SWCNT and the s-SWCNT/Cyt c nanohybrid solutions, respectively. **d**, Schematic diagrams of s-SWCNT/Cyt c nanohybrid films (upper) and the band-edge offset across the s-SWCNT/Cyt c interface (lower) for exciton dissociation. **e**, Diagram of the IR photodetector device schematic on SiO_2_/Si substrate with two Au/Ti electrodes.

Figure 1a depicts schematically an s-SWCNT/Cyt c nanohybrid building block with a Cyt c molecule wrapping around an s-SWCNT via self-assembly in solution prior to film formation (see Methods for details). Atomic force microscopy (AFM) was applied to characterize the morphology of the s-SWCNT/Cyt c building blocks dispersed on SiO_2_/Si substrates. Figure 1b shows a representative AFM image of several s-SWCNT/Cyt c building blocks with the length in the range of 0.3–5.0 μm and the diameter of 2.2 to 3.0 nm. The diameter of the s-SWCNT/Cyt c hybrid building block is obviously larger than that of the original s-SWCNTs in the range of 1.2–1.7 nm. Considering the Cyt c is a small molecule, primarily a chain of amino acids of small diameter typically on the order of 1–3 nm or less, the measured diameter of the s-SWCNT/Cyt c hybrid building block is consistent with the anticipated Cyt c absorbing to and wrapping around the individual s-SWCNTs, which is further confirmed by high-resolution electron microscope (HRTEM) images (Fig. S1 in Supplemental Information). The small diameter of the s-SWCNT/Cyt c hybrid building blocks allows much higher SWCNTs concentration in the s-SWCNT/Cyt c thin films. At the 150−200 nm film thickness selected for this work (see film morphology analyzed using scanning electron microscopy (SEM) and HRTEM in Fig. S1a,b in Supplemental Information), the effective SWCNTs thickness could be up to 80–110 nm, which is close to the optimal SWCNTs thickness for an almost complete NIR absorption^11^.

Figure 1c compares the optical absorbance spectra of the pure Cyt c and the s-SWCNT/Cyt c nanohybrid suspensions measured using a Cary 5000 ultraviolet-visible-NIR dual-beam spectrophotometer. The UV-visible absorption spectra were measured by fixing a concentration of Cyt c (100 μg/ml) and s-SWCNT (50 μg/ml). Evidently, these absorption peaks of the s-SWCNT/Cyt c nanohybrid at 408, 522 and 550 nm come from Cyt c. The broad absorption peak in the wavelength range of 900 to 1300 nm is attributed to the first interband transitions of s-SWNTs, and the sharpness and strength of absorption peak depends on the semiconducting purity of SWCNTs^20^,^24^. In particular, the pure Cyt c is almost transparent at above ~600 nm wavelength. This means the s-SWCNTs will dominantly contribute to the NIR absorption and photoexciton under NIR illumination. As depicted in Figs. 1d and S1 (in Supplementary Information), the s-SWCNT/Cyt c nanohybrid film can be viewed as a composite or network of well dispersed s-SWCNT/Cyt c building blocks, in each of them a heterojunction is formed across the s-SWCNT/Cyt c interface with SWCNT and Cyt c serving, respectively, as electron donor and acceptor according to their band edge alignment. These heterojunctions play a critical role in exciton dissociation into free charge carriers upon NIR photon absorption by s-SWCNTs. In fact, this heterojunction composite structure is similar to bulk inorganic/organic heterojunction structure employed in organic solar cells for efficient exciton dissociation and hence large photocurrent generation^27^,^28^. The s-SWCNT/Cyt c nanohybrid composite, however, has an unique advantage in terms of much high concentration of the light absorber (SWCNT in this case) achievable through selection of small pairing molecules such as Cyt c. Furthermore, the highly efficient electron (or hole) transport properties of the biomolecules are essential to facilitate charge transport through the composite with minimal charge recombination. In this regards, Cyt c is an excellent choice for s-SWCNT to form efficient electron/hole transport in the s-SWCNT/Cyt c nanohybrid with s-SWCNT and Cyt c serving as hole and electron transport channels respectively. Figure 1e shows schematically the s-SWCNT/Cyt c nanohybrid device employed in this work for characterization of NIR detection. When light impinges onto the photodetector, excitons are generated in the s-SWCNTs and dissociated at the s-SWCNT/Cyt c heterojunctions into free carriers, resulting in enhanced photoconductivity as the photoresponse.

**Figure 2:**
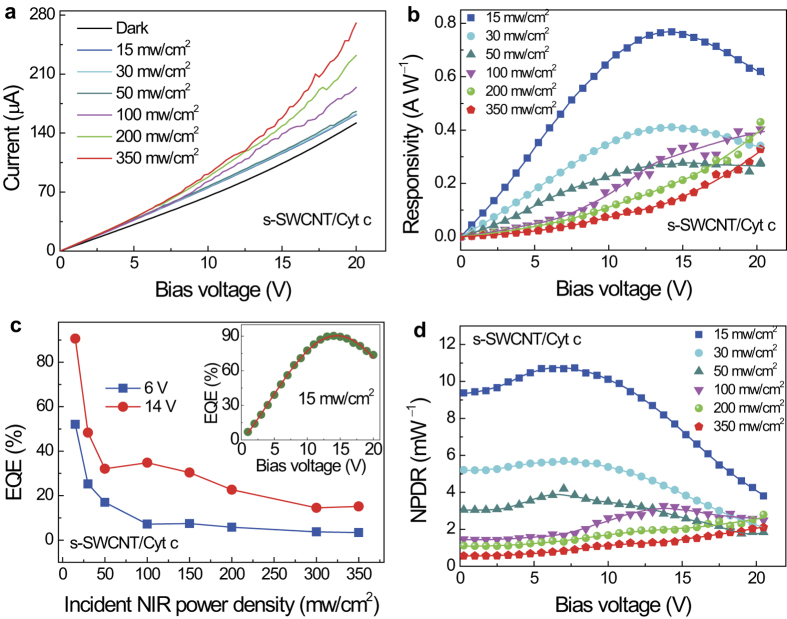
Photoresponse and external quantum efficiency of the s-SWCNT/Cyt c nanohybrid. **a**, Electric current of the s-SWCNT/Cyt c devices under dark and NIR illumination as a function of bias voltage at various incident NIR power density from 15 to 350 mW/cm^2^. **b**, Bias voltage dependence of the photoresponsivity at various incident NIR power density from 15 to 350 mW/cm^2^. **c**, External quantum efficiency as a function of incident NIR power density. The inset shows the quantum efficiency versus bias voltage under 15 mW/cm^2^ NIR light. **d**, The normalized photocurrent-to-dark current ratio (NPDR) as function of the bias voltage.

Figure 2a illustrates the *V−I* characteristic measured on a representative s-SWCNT/Cyt c photodetector in response to NIR illumination with various incident power densities ranging from 15 to 350 mW/cm^2^. Accordingly, the effective incident NIR power (*P*_in_) can be calculated by the NIR power density multiplying by the irradiated sample area. A strong photoresponse is clearly demonstrated. In particular, the *V−I* curves are highly nonlinear in a similar way to the s-SWCNT/P3HT nanohybrid case^16^. This nonlinear *V−I* characteristic differs fundamentally from the linear one reported in CNT-based bolometers^14^,^15^ and is anticipated from the heterojunctions implemented for exciton dissociation to photocurrent, instead of heat. In order to quantify the photoresponse, photoresponsivity *R*_*i*_ defined as photocurrent-to-incident NIR power ratio (*I*_photo_/*P*_in_) is calculated and compared in Fig. 2b with respect to different NIR intensities. Two different types of behaviors are evident. At low NIR light intensity before 100 mW/cm^2^, the *R*_*i*_ increases with the bias voltage initially, followed with a reversed trend after its saturation at a peak *R*_*i*_ value located in the bias voltage range of 14 −16 V. The peak *R*_*i*_ values are 0.77, 0.41 and 0.28 A W^−1^ at NIR light intensities of 15, 30, and 50 mW/cm^2^, respectively. These high *R*_*i*_ values observed on the s-SWCNT/Cyt c photodetectors are more than two orders of magnitude higher than that of their s-SWCNT/P3HT counterparts and represents the best so far achieved on the CNT-based IR detectors^14^,^15^,^16^,^18^,^19^,^20^,^21^. It is worth mentioning that the high *R*_*i*_ obtained in the s-SWCNT/Cyt c photodetectors is comparable to and slightly better than the recent reports on waveguide-integrated graphene NIR photodetectors^29^−^31^. This enhancement could be attributed to the dramatically improved EQE defined as the ratio of the number of the photo-generated charges to the number of incident photons^32^: EQE = *hνI*_photo_/*eP*_in_ = *hνR*_i_/*e*, where *h* is the Planck’s constant, *ν* is the optical frequency and *e* is the el*e*ctron charge. As shown in Fig. 2c, the EQE is up to 90.5% in the case of the s-SWCNT/Cyt c nanohybrids in contrast to only 1.72% in their s-SWCNT/P3HT counterparts primarily because a much higher SWCNT concentration by more than an order of magnitude in the former. In addition, improved charge transport through the Cyt c molecular chains, as compared to charge hoping in bulk P3HT with extremely short diffusion length for holes (P3HT is a hole acceptor in the s-SWCNT/P3HT nanohybrid), may add on further contributions to the enhanced EQE and *R*_*i*_ in the s-SWCNT*/*Cyt c NIR detectors.

The trend of monotonic decreasing *R*_*i*_ values with increasing NIR power intensity at a given bias voltage in the range of 0–20 V, correlates well with the trend of decreasing EQE with increasing NIR light intensity shown in Fig. 2c. At higher NIR light intensities, the *R*_*i*_ peak disappears as a consequence of monotonic increase of the *R*_*i*_ w_*i*_th increasing bias voltage. The overall *R*_*i*_ as well as EQE at higher NIR intensities are considerably lower than that in the lower intensity range. The EQE also depends on the bias voltage at a given incident NIR power intensity and the highest EQE was observed at around 14 V bias voltage under NIR power intensity of 15 mW/cm^2^ (inset of Fig. 2c).

To shed some lights on the contribution of the Cyt c in the measured photoresponse in the s-SWCNT/Cyt c devices, the same measurement as detailed in Fig. 2 on the s-SWCNT/Cyt c devices was repeated on pure Cyt c devices of a similar geometry. Several fundamental differences have been observed between the pure Cyt c (Fig. S2 in Supplementary Information) and s-SWCNT/Cyt c hybrid devices (Fig. 2). First, the photoresponse in the former is significantly smaller than that in the latter by at least one order of magnitude depending on the NIR light intensity. On the other hand, the NIR power intensity dependence of the *R*_i_ is qual_i_tatively different in these two types of devices. Specifically, the *R*_i_ of the pure Cyt c device is negligibly small at low NIR light intensity below 50 mW/cm^2^ illumination power intensity (Figs. S2a and S2b in Supplementary Information), in contrast to the higher *R*_i_ values at lower NIR power intensity in the s-SWCNT/Cyt c hybrid devices. This results in significantly different EQE vs. NIR power intensity curves in these two types of devices as shown in Figs. 2c and S2c, suggesting different mechanisms responsible for the NIR phontoresponse in these two cases. In fact, bolometric effect was reported in Cyt c photodetectors previously^33^,^34^. Nevertheless, a significant contribution of such a bolometric effect from pure Cyt c to the NIR photoresponse in s-SWCNT/Cyt c hybrid NIR detectors is unlikely considering the *R*_i_ as high as 0.77 A W^−1^ at NIR power intensity of 15 mW/cm^2^ in the hybrid devices is at least two orders of magnitude larger than that of the pure Cyt c control devices. Furthermore, the EQE in the Cyt c control devices is very low and the maximum observed value is ~2.2% (Fig. S2c in Supplementary Information). These results therefore confirm that the efficient exciton dissociation into photocurrent at the s-SWCNT/Cyt c heterojunctions and the efficient charge transport along the Cyt c chains are the key to high photoresponsivity in the s-SWCNT/Cyt c hybrid NIR detectors.

To gain further insights in the performance of the photodetectors, the ratio of photocurrent to dark current (*I*_photo_/*I*_dark_) is calculated. Since the incident NIR power *P*_in_ have a considerable effect on the *I*_photo_, the normalized photocurrent-to-dark current ratio defined as NPDR = (*I*_photo_/*I*_dark_)/*P*_in_ = *R*_i_/*I*_dark_ is used as a more objective parameter to eliminate effect of the *P*_in_. The NPDR vs. bias voltage curves of the s-SWCNT/Cyt photodetector at different incident NIR power intensity is depicted in Fig. 2d. A general trend of higher NPDR at lower NIR power intensity can be clearly seen, which suggests a larger specific portion of the dark current generation at higher NIR intensity due to most probably of the heating by incident light. Nevertheless, it is remarkable that NPDR value as high as ~10.8 mW^−1^ can be achieved on the s-SWCNT/Cyt c photodetectors, which represents an improvement of approximately two orders of magnitude with respect to the case of the s-SWCNT/P3HT nanohybrid NIR detectors^16^. In fact, this performance is on par with the reported value of 10 mW^−1^ on the waveguide-integrated graphene IR detectors^30^.

**Figure 3:**
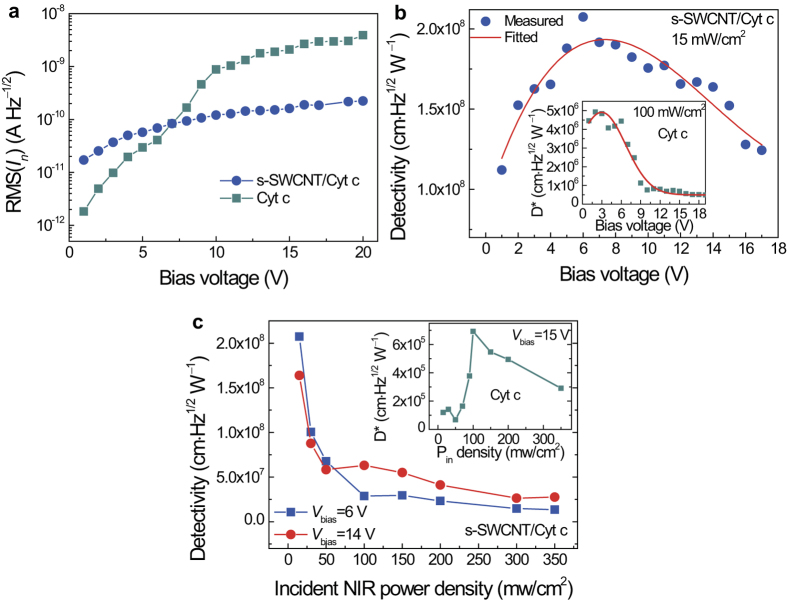
Noise and detectivity. **a**, Comparison of root-mean-square noise currents for nanohybrid s-SWCNT/Cyt c and pure Cyt c devices at different bias voltages. **b**, Bias voltage dependence of detectivity of nanohybrid s-SWCNT/Cyt c under incident NIR power density of 15 mW/cm^2^. The inset shows the best detectivity of the pure Cyt c devices versus bias voltage under incident NIR power density of 100 mW/cm^2^. **c,** Detectivity at 6 and 14 V bias voltage as functions of incident NIR power density. The inset plots the detectivity of the pure Cyt c devices versus incident NIR power density.

An important parameter related to the sensitivity of a photodetector is the noise equivalent power (NEP), which represents the incident light power required for the detector output signal to be equal to the noise current, typically expressed with units of W Hz^−1/2^. NEP is defined by NEP = 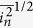/*R*_i_ where the 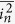 is the mean square noise current calculated from the spectra density of noise power. The 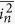 of the s-SWCNT/Cyt c and the pure Cyt c devices monotonically decreases with increasing frequency, which can be fitted by 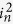
∝1/*f* in the low frequency range up to few kHz, indicating that 1/*f* noise dominates the current noise behavior in at low frequencies (Figs. S3a and S4a in Supplementary Information). Figure 3a reveals a monotonic, linear bias voltage dependence of root mean square noise current RMS(*I*_n_), 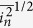 of the s-SWCNT/Cyt c nanohybrid devices and its magnitude spans across the range of 1.7 × 10^−11^–2.2 ^× ^10^−10 ^ AHz^−1/2^ in the bias voltage range of 1–20 V (Blue circle). This compares well with the reported noise characteristic in CNTs and suggest the CNTs dominate the noise spectra in the s-SWCNT/Cyt c nanohybrids^15^,^16^. The calculated NEP is plotted against the bias voltage. The minimum NEP value of ~1.5 × 10^−10^ W Hz^−1/2^ is observed at ~6 V bias under 15 mw/cm^2^ (Fig. S3b in Supplementary Information). While the NEP increases with either increasing or decreasing bias voltage at higher or lower bias voltages. Interestingly, the NEP of the pure Cyt c control device presents an exponential increase with increasing bias and is ~3 orders of magnitude larger than that of the s-SWCNT/Cyt c device (Fig. S4b in Supplementary Information).

The differences in NEP lead to different figure-of-merit detectivity D* that is independent of the device area in characterizing photodetector’s performance, in these two types of the devices. The D* can be calculated by D* = (*A*)^1/2^/NEP = *R*_i_(*A*)^1/2^/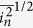, where *A* is the detection area with unit of cm^2^. On the s-SWCNT/Cyt c hybrid device, the best D* is as high as ~2 × 10^8  ^cm Hz^1/2^W^**−**1^ at the bias voltage of ~ 6 V under 15 mW/cm^2^, and the trend in D* vs. bias voltage curve (Fig. 3b) correlates well with the bias dependence of *R*_*i*_ and 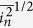 (Figs. 2b and 3a). While this is comparable to the best D* values reported for uncooled CNT-based IR detectors^16^, higher D* could be projected at lower NIR power intensity. In contrast, the D* of the pure Cyt c control device is about two orders of magnitude lower (inset in Fig. 3b), which is not surprising considering its lower responsivity and higher noise (Figs. S2b and S4b in Supplementary Information). Fig. 3c shows the D* of s-SWCNT/Cyt c and the pure Cyt c devices as functions of incident NIR power density. It is noted that the D* of s-SWCNT/Cyt c monotonically decreased with increasing incident NIR power, which implies our devices have an advantage of high sensitivity in low energy photon detection. This is essentially different from the pure Cyt c whose D* shows a parabola law and appears a peak at 100 mW/cm^2^ due to their different NIR phontoresponse mechanisms, which is consistent with the dependence of the EQE versus light intensity.

**Figure 4:**
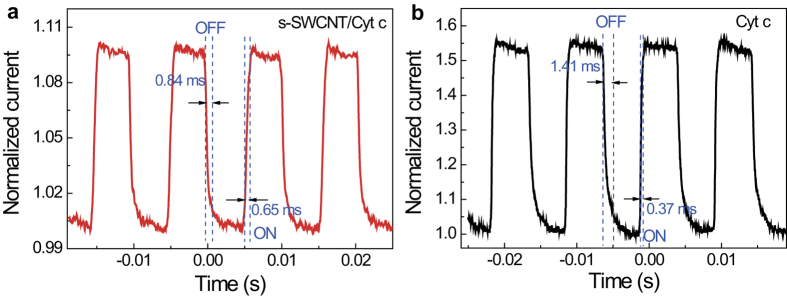
Dynamic photoresponse. Representative dynamic photoresponse measured on **a**, the s-SWCNT/Cyt c nanohybrid and **b**, the pure Cyt c devices at NIR modulation frequency of 97 Hz.

Figure 4 compares the dynamic photoresponse of the s-SWCNT/Cyt c and the pure Cyt c devices at 97 Hz NIR modulation frequency. The response times of the s-SWCNT/Cyt c nanohybrid device for light on and off are 0.65 ms and 0.84 ms, respectively, which are calculated from 10% to 90% maximum photocurrent. It is worth noting that the photoresponse of the s-SWCNT/Cyt c device is considerably faster than that of the pure SWCNT devices, or in general the CNT-based IR bolometers^14^,^15^,^16^,^18^,^19^,^20^,^21^. While the observed response times are comparable to that on s-SWCNT/P3HT nanohybrid NIR detectors, some remarkable differences exist. In particular, the time constant of 0.84 ms corresponding to the light off in the s-SWCNT/Cyt c devices is considerably shorter than the 1.4 ms of s-SWCNT/P3HT nanohybrid NIR detectors. Considering the two types of detectors have a similar light-on time constant in the range of 0.6–0.65 ms, the temporal responses to NIR light on and off are much more symmetric in the s-SWCNT/Cyt c devices as compared to their s-SWCNT/P3HT counterparts. This symmetry means not only better photodetector performance, but also the reduced charge trapping effect in the former. While a thorough understanding of the microscopic charge transport mechanism in s-SWCNT/Cyt c hybrid requires substantial future works, we propose a hypothesis that the slower charger transport in Cyt c as opposed to the faster one in SWCNT in the s-SWCNT/Cyt c nanohybrids bottlenecks the response time. This argument is supported by the comparable light-on and light-off time constants observed in the pure Cyt c control devices illustrated in Fig. 4b. If a similar argument would be applied to the case of s-SWCNT/P3HT nanohybrids, the improved light-off response time in the s-SWCNT/Cyt c nanohybrids may be attributed to the considerably better charge transport medium of Cyt c than P3HT.

## Conclusions

We have developed a novel s-SWCNT/Cyt c nanohybrid for uncooled infrared detection. The demonstrated higher photoresponsivity by more than two orders of magnitude than the best reported on CNT-based IR detectors due to the high EQE in exceeding 90% illustrates the importance and feasibility of material design at molecular level in nanohybrids. This nanohybrid approach is attractive for high-performance and low-cost optoelectronic applications because it allows: 1) molecular-scale design of material building blocks that can have light-solid interactive properties superior to conventional materials^35^,^36^, 2) large-scale device fabrication with compatibility to existing microfabrication procedures, and 3) on-chip integration with Si-based readout circuits.

## Methods

### Fabrication of the s-SWCNT/Cyt c films photodetector

Firstly, the suspensions of Cyt c and s-SWCNT were prepared separately. The Cyt c (purity ≥ 95%) from equine heart was directly dissolved in deionized (DI) water to form a solution of 2 mg/ml. The s-SWCNTs (purity of semiconducting SWCNTs ~95% with diameters ranging from 1.2 to 1.7 nm and a length distribution from 300 nm to 5 μm) with surfactant triton were also dissolved in DI water to form a SWCNTs suspension with concentration of ~5 μg/ml. The two suspensions were mixed at 1:40 (SWCNTs: Cyt c) mass ratio and the mixture was kept in an ice-water bath and sonicated (Branson1800) for 3 h. After sonication, the samples were centrifuged to remove insoluble material. For s-SWCNT/Cyt c film fabrication, 0.2 micron mixed cellulose ester (MCE) filter membranes were employed in a vacuum filtration apparatus. Care was taken in transferring the s-SWCNT/Cyt c solution into the filter funnel to avoid bubbles on the solution surface. Bursting surfactant bubbles disrupted the film continuity when it was wet and fragile. The thickness of the formed films is 150*–*200 nm. Two Au(40 nm)/Ti(4 nm) electrodes with spacing of 0.3–0.4 mm were pre-deposited onto the SiO_2_/Si substrates using electron-beam evaporation through a shadow mask. s-SWCNT/Cyt c film with 0.2–0.4 mm width is transferred onto the substrate with the pre-deposited Au/Ti electrodes followed with dissolving the filtration membrane. The pure Cyt c film devices (channel width and length are 2–2.5 mm and 0.3–0.4 mm, respectively) were fabricated on the same substrate with the same electrodes as a control sample by using a prepared solution of 2 mg/ml.

### Photoresponse measurements

All measurements were carried out at room temperature and under atmospheric conditions. To measure the photocurrent in accordance with the voltage source mode, the measured circuit was set up that the device was connected in series with a constant resistor. Bias voltage was applied in the circuit using an Agilent E3631A voltage source, and the electric current was determined using a HP 34420A voltmeter. The illumination was provided by an xenon light with a NIR filter for the passing band of 1.0*–*1.3 μm. The incident light power density was calibrated using a Thorlabs PM100D thermal power meter. Dynamic photoresponse at various modulation frequencies controlled by a mechanical chopper was measured using an Agilent 54624A oscilloscope. The noise spectra at various bias voltages were obtained using a Stanford Research SR760 spectrum analyzer and an Agilent E3631A voltage source.

## Additional Information

**How to cite this article**: Gong, Y. *et al.* Wrapping cytochrome c around single-wall carbon nanotube: engineered nanohybrid building blocks for infrared detection at high quantum efficiency. *Sci. Rep.* **5**, 11328; doi: 10.1038/srep11328 (2015).

## Supplementary Material

Supplementary Information
