# Supplementary material for: Wrapping cytochrome c around single-wall carbon nanotube: engineered nanohybrid building blocks for infrared detection at high quantum efficiency
Source: Sci Rep. 2015 Jun 11;5:11328. doi: 10.1038/srep11328 (PMC4463958; doi:10.1038/srep11328)
Supplement: Supplementary Information [file srep11328-s1.doc]

Wrapping cytochrome c around single-wall carbon nanotube: engineered nanohybrid building blocks for infrared detection at high quantum efficiency

Youpin Gong^1^, Qingfeng Liu^1^, Jamie Samantha Wilt^1^, Maogang Gong^2^, Shenqiang Ren^2^ & Judy Wu^1^

^1^*Department of Physics and Astronomy, University of Kansas, Lawrence, Kansas, 66045,* *USA*, ^2^*Department of Chemistry, University of Kansas, Lawrence, Kansas 66045, USA*.

Correspondence and requests for materials should be addressed to Y.P.G. (ygong@ku.edu) or J.W. (jwu@ku.edu).


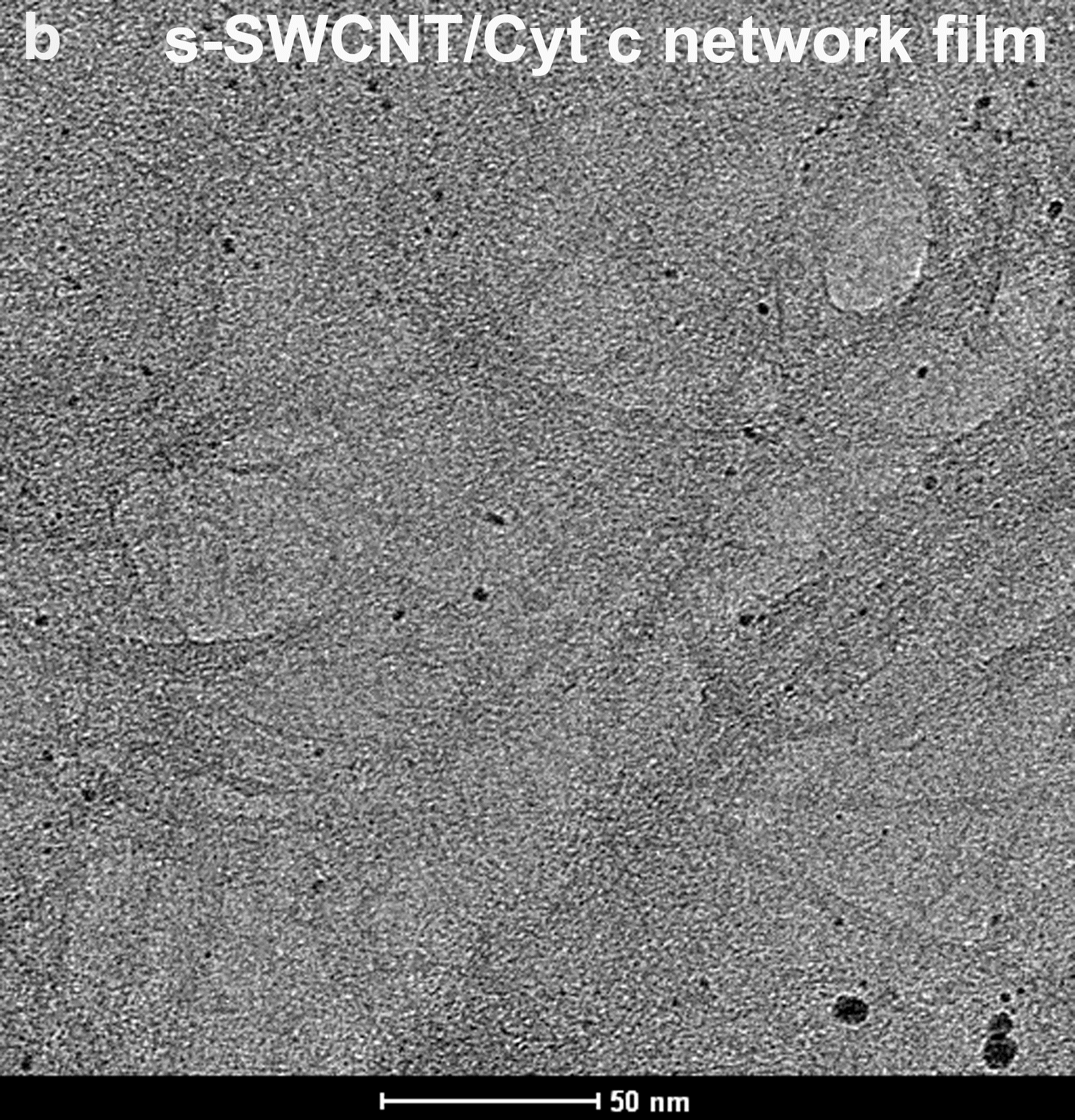

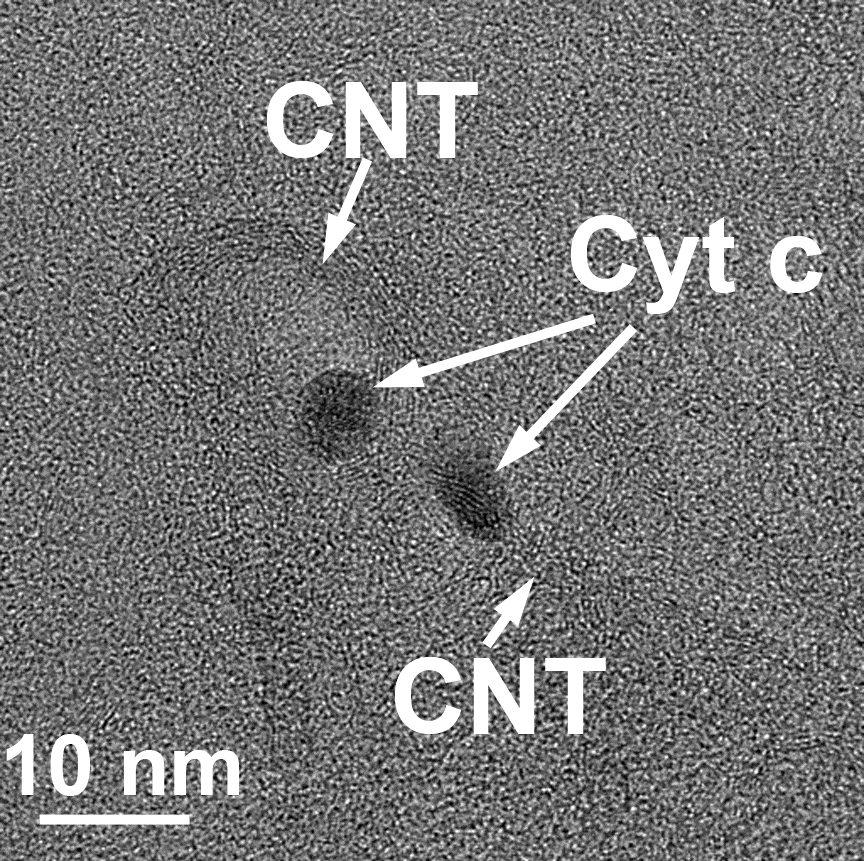

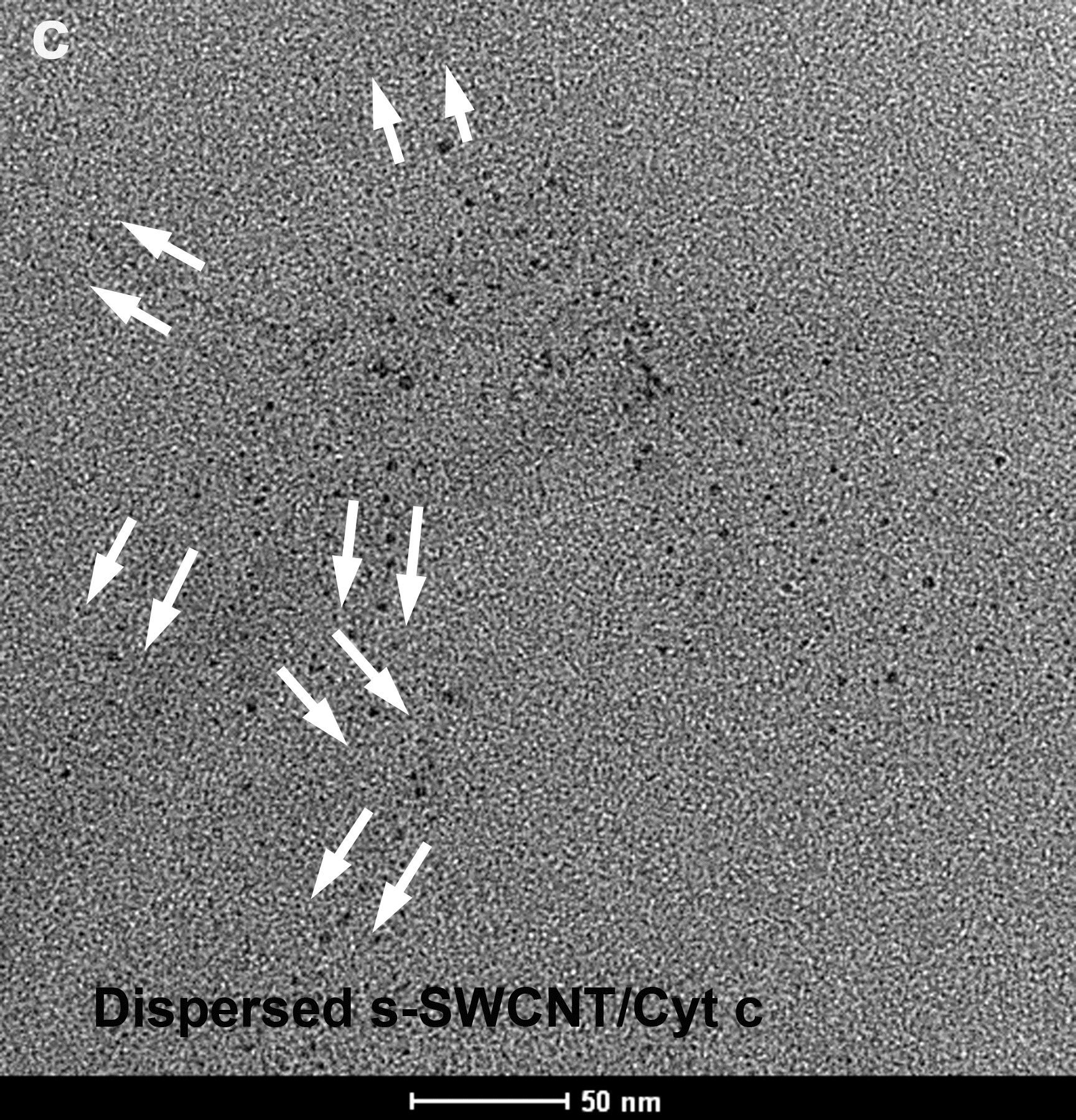

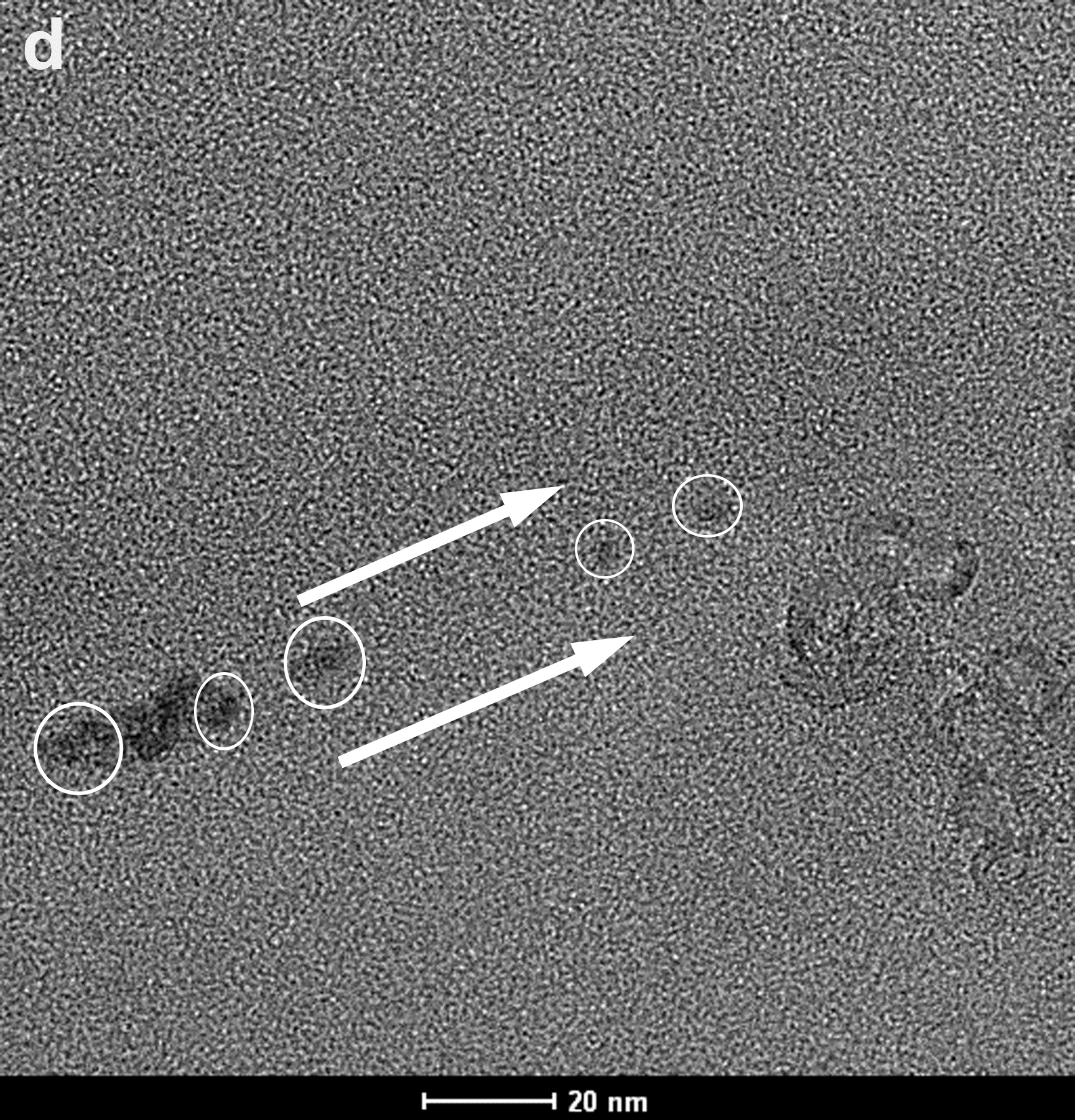

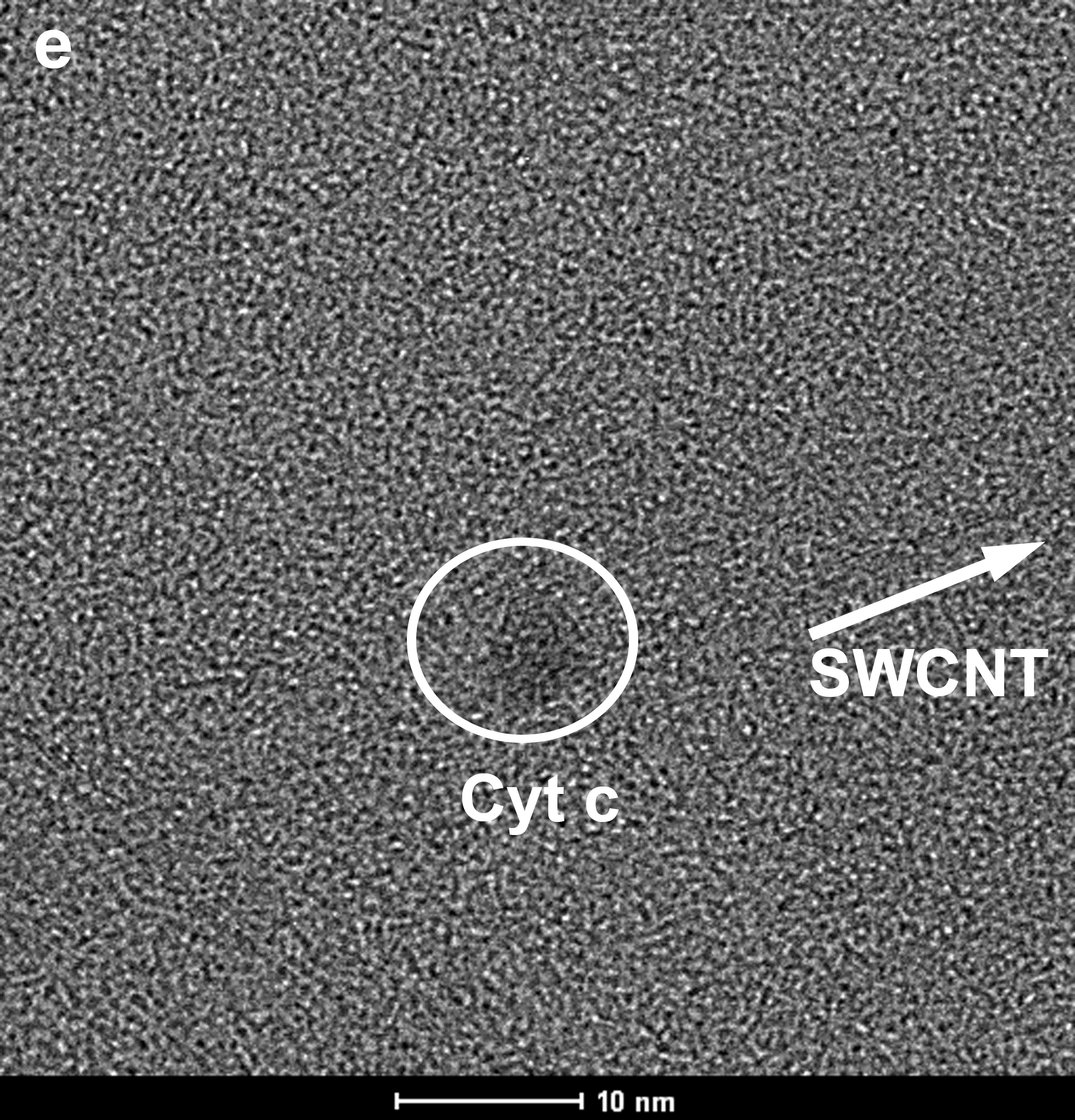

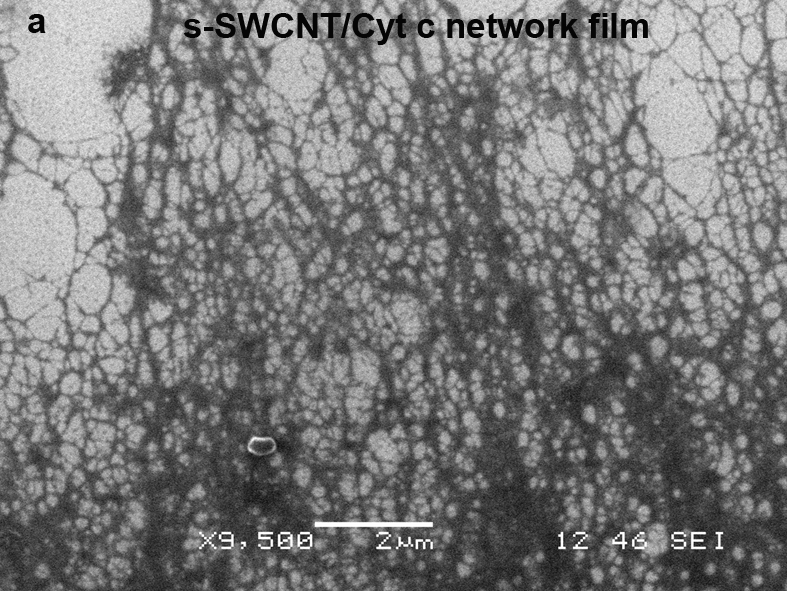


**Figure S1. a**,**b**, SEM (**a**) image of an s-SWCNT/Cyt c network film on SiO_2_/Si surface and TEM (**b**) image s-SWCNT/Cyt c network film on Cu micro grid. Inset in **b** shows a detail of Cyt c absorbed by the SWCNTs. **c**, Low magnification TEM image shows that some Cyt c wrap on the dispersed s-SWCNTs along the wall. A s-SWCNT/Cyt c is marked in the middle of two white arrow. **d**,**e**, HRTEM images of s-SWCNT/Cyt c zooming in on typical regions in **c** represent that the Cyt c molecules wrap (or absorb) around an SWCNT.

**Figure S2. a**, *I*-*V* curves of the pure Cyt film devices under dark and NIR illumination of various incident NIR power density from 15 to 350 mW/cm^2^. **b**, Bias voltage dependence of the photoresponsivity of the pure Cyt c film devices at various incident NIR power density from 15 to 350 mW/cm^2^. **c**, EQE of the pure Cyt c film device as a function of incident NIR power density.

**Figure S3.** **a**, Spectra of current noise power density of s-SWCNT/Cyt c nanohybrid in the double logarithmic coordinates, showing 1/*f* noise dominate the current noise behavior in the low NIR modulation frequency range. **b**, Noise equivalent power NEP as function of the bias voltage taken on the s-SWCNT/Cyt c devices.

**Figure S4.** **a,** Spectra of current noise power density of the pure Cyt c device in the double logarithmic coordinates. **b,** NEP versus bias voltage of the pure Cyt c devices.
